# Organic–Inorganic Hybrid Cuprous‐Based Metal Halides for Warm White Light‐Emitting Diodes

**DOI:** 10.1002/advs.202203596

**Published:** 2022-09-06

**Authors:** Xuan Meng, Sujun Ji, Qiujie Wang, Xiaochen Wang, Tianxin Bai, Ruiling Zhang, Bin Yang, Yimeng Li, Zhipeng Shao, Junke Jiang, Ke‐li Han, Feng Liu

**Affiliations:** ^1^ Institute of Molecular Sciences and Engineering Institute of Frontier and Interdisciplinary Science Shandong University Qingdao 266237 P. R. China; ^2^ State Key Laboratory of Molecular Reaction Dynamics Dalian Institute of Chemical Physics Chinese Academy of Science Dalian 116023 P. R. China; ^3^ Qingdao Institute of Bioenergy and Bioprocess Technology Chinese Academy of Sciences Qingdao 266101 P. R. China; ^4^ ISCR (Institut des Sciences Chimiques de Rennes)‐UMR CNRS 6226 ENSCR, Université de Rennes Rennes 35700 France

**Keywords:** cuprous‐based phosphors, electroluminescence application, hybrid metal halides, single‐component warm white‐light emitters, warm white light‐emitting diodes (LEDs)

## Abstract

Single‐component emitters with stable and bright warm white‐light emission are highly desirable for high‐efficacy warm white light‐emitting diodes (warm‐WLEDs), however, materials with such luminescence properties are extremely rare. Low­dimensional lead (Pb) halide perovskites can achieve warm white photoluminescence (PL), yet they suffer from low stability and PL quantum yield (PLQY). While Pb‐free air‐stable perovskites such as Cs_2_AgInCl_6_ emit desirable warm white light, sophisticated doping strategies are typically required to increase their PL intensity. Moreover, the use of rare metal‐bearing compounds along with the typically required vacuum‐based thin‐film processing may greatly increase their production cost. Herein, organic–inorganic hybrid cuprous (Cu^+^)‐based metal halide MA_2_CuCl_3_ (MA = CH_3_NH_3_^+^) that meets the requirements of i) nontoxicity, ii) high PLQY, and iii) dopant‐free is presented. Both single crystals and thin films of MA_2_CuCl_3_ can be facilely prepared by a low‐cost solution method, which demonstrate bright warm white‐light emission with intrinsically high PLQYs of 90–97%. Prototype electroluminescence devices and down‐conversion LEDs are fabricated with MA_2_CuCl_3_ thin films and single crystals, respectively, which show bright luminescence with decent efficiencies and operational stability. These findings suggest that MA_2_CuCl_3_ has a great potential for the single‐component indoor lighting and display applications.

## Graphical abstract

The newly developed hybrid MA_2_CuCl_3_ meets the requirements of i) broadband warm white‐light emission, ii) nontoxicity, iii) high photoluminescence quantum yield, iv) dopant‐free, v) low‐cost, and vi) excellent film‐forming ability. Besides, the first successful electroluminescence application of MA_2_CuCl_3_ opens a new avenue toward single‐component warm white light‐emitting diodes.

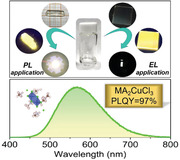

## 1 Introduction

High‐performance warm white light‐emitting diodes (warm‐WLEDs) are highly required for the widespread use of solid‐state lighting especially indoors.^[^ ^1^, ^2^ ^]^ There are currently three main methods for producing warm‐WLEDs: first, coating yellow and red phosphors on blue LEDs; second, coating a mixture of red, green, and blue (RGB) phosphors on UV LEDs; third, directly mixing RGB LEDs. However, each of these three technologies has their own shortcomings, such as the poor color rendition, low emission efficiency, and discontinuity in the visible light spectrum.^[^ ^3^, ^4^ ^]^ Development of new single‐component warm white light‐emissive phosphors with high photoluminescence quantum yield (PLQY) and color stability bears great significance for high‐performance warm‐WLEDs: On the one hand, single‐component warm white phosphors do not rely on additional red phosphors to adjust their correlated color temperature (CCT); meanwhile, because of the unique broad‐spectrum luminescence characteristics, warm white phosphors can solve the problem of discontinuity in the visible light spectrum of RGB LEDs. On the other hand, single materials with efficient and stable warm white‐light emission can be directly fabricated into electrically driven warm‐WLEDs, which ultimately addresses concerns of the aforementioned down‐conversion applications.^[^ ^2^, ^5^, ^6^, ^7^ ^]^

However, there are very few compounds of the nonrare earth ions which have high‐efficiency warm white emission. Highly distorted low‐dimensional lead (Pb)‐based perovskites such as 2D *α*‐(DMEN)PbBr_4_ (DMEN = 2‐(dimethylamino)ethylamine) are found to be capable of emitting warm white light.^[^ ^8^ ^]^ However, the inherent toxicity of Pb will hamper their practical commercialization; Pb‐free double perovskites such as 3D Cs_2_AgInCl_6_ and some pure In‐based 0D perovskites (e.g., Cs_2_InCl_5_·H_2_O) are also found to possess warm white fluorescence, but optical properties of these halide derivatives are highly sensitive to trace element doping, which brings difficulty to large‐scale production and reproducibility. Besides, indium belongs to costly rare earth element, which hampers widespread employment of these materials.^[^ ^6^, ^9^, ^10^, ^11^ ^]^ 0D tin (Sn)‐halide perovskites are also commonly studied as potential warm white‐light emitters, yet significant challenges remain as Sn^2+^ can be easily oxidized to Sn^4+^ upon air exposure.^[^ ^12^, ^13^ ^]^ In recent years, cuprous (Cu^+^)‐based metal halides have received extensive attention in the research field of luminescence because they are nontoxic, earth‐abundant, and intrinsically highly emissive without the aid of any sophisticated doping. For example, A_2_CuX_3_ (A = K, Rb; X = Cl, Br), MA_4_Cu_2_Br_6_ (MA = CH_3_NH_3_^+^), (TBA)CuX_2_ (TBA = tetrabutylammonium cation), and Cs_3_Cu_2_X_5_ (X = Cl, Br, I) can be facilely prepared via solution synthesis, which show near‐UV to green emission with near‐unity PLQYs.^[^ ^14^, ^15^, ^16^, ^17^, ^18^, ^19^, ^20^ ^]^ In addition to these narrowband emissions, the broadband emission which is a prerequisite for yielding warm white light can be also achieved with these Cu^+^‐based compositions, such as CsCu_2_I_3_, [KC_2_]_2_[Cu_4_I_6_] (C = 12‐crown‐4 ether), [(C_3_H_7_)_4_N]_2_Cu_2_I_4_, (C_16_H_36_N)CuI_2_, (Gua)_3_Cu_2_I_5_ (Gua = guanidine), etc.^[^ ^21^, ^22^, ^23^, ^24^, ^25^ ^]^ However, despite the broadband emission, very few of these materials exhibit the desirable warm white light which features CCT value of around 2700 to 4000 K. Besides, one can see that most of these Cu^+^‐based broadband emissive halides are iodide compounds, which severely restricts the possible adjustment of the spectral response through halide composition. The aforementioned facts highlight the urgency to develop new single‐component phosphors which have merits of broadband warm white‐light emission, low‐cost processing, nontoxicity, high PLQY (without extrinsic doping), and high color stability.

In this work, an efficient ultrabroad‐band solid warm white‐light emission from dopant‐free Cl‐based metal halide MA_2_CuCl_3_ is first reported. MA_2_CuCl_3_ can be facilely prepared in the form of centimeter‐sized single crystals, which show desirable PL emission in the visible range from 450 to 800 nm, CCT of 3607 K, and an intrinsically high PLQY of ≈97%. Spectroscopic characterizations reveal an exceptionally strong exciton–phonon coupling in MA_2_CuCl_3_, which surpasses most other Cu^+^‐based metal halides. Theoretical calculations further unveil a unique electronic structure for MA_2_CuCl_3_, which features highly localized holes and spatially isolated excitons. These correlate well with their high PLQYs and long radiative lifetimes. To better utilize the intrinsically high PLQYs of MA_2_CuCl_3_ and to enhance their prospects and potential in warm‐WLEDs, we further devised a novel methanol‐based approach to deposit large‐scale and uniform MA_2_CuCl_3_ thin films, which show high PLQY of ≈90%. Compared to traditional methods that employ coordinating solvents, such as *N*,*N*‐dimethylformamide (DMF) and dimethylsulfoxide (DMSO), the methanol‐produced thin films are of higher quality in terms of continuity and compactness. The ensuing electrically driven LEDs based on MA_2_CuCl_3_ thin films achieve a maximum luminance of 54 cd m^−2^ and a maximum external quantum efficiency (EQE) of 0.035%, which is comparable to that of state‐of‐the‐art warm white‐emissive Pb‐free perovskite LEDs; UV‐pump LEDs based on MA_2_CuCl_3_ solids show a brightness up to 5500 cd m^−2^ and a maximum EQE of 0.6%, which retain >90% of the initial luminance after 45 min of operation in ambient air. Our findings unambiguously show that the hybrid metal halide MA_2_CuCl_3_ holds great potential to be used as a new kind of single‐component light emitter in warm‐WLEDs.

## 2 Results and Discussion

**Figure 1:**
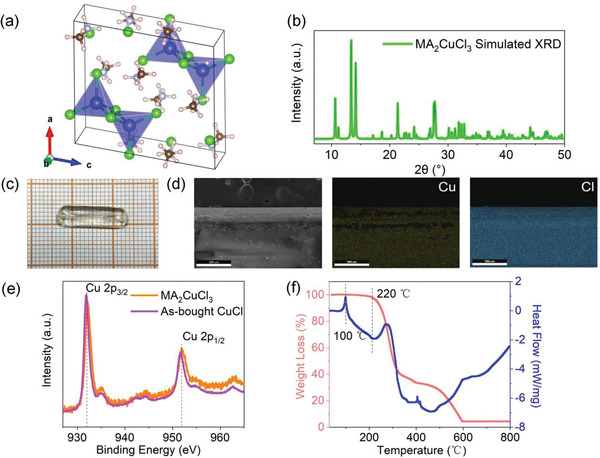
a) Crystal structure of MA_2_CuCl_3_, created with VESTA software based on crystallographic information obtained by X‐ray single crystal structural analyses. b) Simulated powder XRD pattern for MA_2_CuCl_3_. Assignments of peaks and a CIF file are provided in the Supporting Information. c) Digital image of a nearly 2 cm single‐crystal MA_2_CuCl_3_ and d) its element distribution analysis by EDS mapping. e) XPS narrow‐scan spectrum of Cu 2p and f) TG‐DSC curves of MA_2_CuCl_3_ single crystals.

We have succeeded in identifying two synthetic routes toward MA_2_CuCl_3_. The first approach involves an inverse temperature crystallization process, which yields single crystals with centimeter‐scale length. Briefly, powder mixtures containing MACl and CuCl were dissolved in solvent mixture of concentrated hydrochloric acid and phosphinic acid (H_3_PO_2_) at 130 °C and then slowly cooled to 10 °C in a thermostat at a cooling rate of 3 °C min^−1^. After ≈48 h of growth, the crystals were taken out of the solution, washed by hexane, and dried with a N_2_ gun (more experimental details can be found in the Supporting Information). Single‐crystal X‐ray diffraction (SCXRD) analyses performed on the as‐grown crystals suggest a specific chemical composition of MA_2_CuCl_3_, which crystallizes in the monoclinic *P2/n* space group and features a 0D structure at the molecular level. In this material, each [Cu_2_Cl_6_]^4–^ dimer is spatially separated by wide band gap organic framework of MA^+^ (see **Figure** **1**a; Tables S1–S6 of the Supporting Information). Figure 1b shows the simulated powder XRD pattern for MA_2_CuCl_3_. MA_2_CuCl_3_ forms single crystals that present as 0.5 cm wide and 1.8 cm long rod (Figure 1c), whose morphology conforms to the underlying symmetry of the crystal lattice. It is noted that size of the obtained single crystals can be increased even further by using a bigger scintillation vial, significantly larger than most of the other Pb‐free perovskites.^[^ ^25^, ^26^, ^27^, ^28^ ^]^ The large‐scale uniform rod morphology lays the foundation for the design of centimeter‐level single‐crystal devices.^[^ ^28^, ^29^ ^]^ It is important to note that the successful application of this method for crystallization depends on the use of H_3_PO_2_, which was added to prevent the oxidation of Cu^+^,^[^ ^17^ ^]^ otherwise nonluminescent Cu^2+^‐contained compounds can be produced (Figure S1, Supporting Information). Similarly, we have also prepared Br‐based single crystals, MA_2_CuBr_3_, which emit bright green light with the addition of H_3_PO_2_ (Figure S2, Supporting Information).

The inductively coupled plasma‐mass spectrometry measured for the obtained crystals confirms the presence of Cu and Cl elements in near stoichiometric ratio of 1:3. These elements are seen to be uniformly distributed in the crystals as displayed in their energy dispersive X‐ray spectroscopy (EDS) elemental mapping image (Figure 1d). To confirm the presence of Cu element in its expected oxidation state, X‐ray photoelectron spectroscopy (XPS) measurement was further carried out. The measured high‐resolution Cu spectrum consists of a pair separated by the Cu 2p spin–orbit splitting (Figure 1e), each of which has well‐resolved single component at 931.9 and 951.7 eV, respectively, exactly matches the XPS Cu 2p spectrum of the as‐bought CuCl powder. These results thus verify the SCXRD analyses and confirm the absence of those undesired Cu^2+^‐species in the as‐prepared sample. To evaluate thermal stability of the prepared crystals, thermogravimetric analysis coupled with differential scanning calorimetry (TG‐DSC) was carried out. It is seen from Figure 1f that the thermal degradation is multistage. The first stage begins at about 220 °C and the crystal loses 60% of its initial mass, corresponds to the degradation of MACl; the second stage which shows a mass loss of ≈36% can be attributed to the degradation of CuCl with a maximum rate near 600 °C. The above TG results agree well with the mass ratio of MACl/CuCl in MA_2_CuCl_3_ crystal and prove that this material would not decompose until 220 °C. Note that the commonly studied MAPbCl_3_ perovskite exhibits similar decomposition temperature.^[^ ^30^ ^]^ However, unlike MAPbCl_3_, we identify MA_2_CuCl_3_ metal halide undergoes a melting process at ≈100 °C, as evidenced by the obvious endothermic peak in its DSC curve (Figure 1f, blue line).

Single crystals of MA_2_CuCl_3_ can be also prepared by room‐temperature solvent evaporation‐induced crystallization strategy, following a procedure modified from a previous published synthesis.^[^ ^17^ ^]^ Typically, CuCl and MACl were dissolved in a mixture of DMF and H_3_PO_2_, followed by slow solvent evaporation in a vacuum drying oven at room temperature (more experimental details can be found in the Supporting Information). XRD measurements performed on hand‐ground powders confirm the success of formation of monoclinic‐phase MA_2_CuCl_3_ (Figure S3, Supporting Information). However, most of the obtained crystalline grains have crystal sizes less than 1 mm, inferior than those obtained by inverse temperature crystallization method.

**Figure 2:**
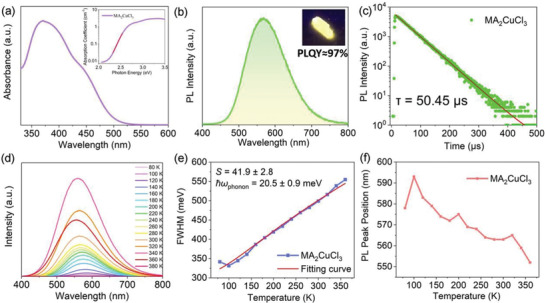
a) UV‐vis absorption spectrum of the MA_2_CuCl_3_ single crystals. Inset shows the exponential spectrum as a function of photon energy, where the straight line indicates the best exponential fit for the tail. b) PL spectrum of the MA_2_CuCl_3_ single crystals, which shows negligible overlap with the absorption spectrum. Inset shows photograph of a typical crystal taken under 254 nm UV light. c) TRPL of the MA_2_CuCl_3_ single crystals. d) Temperature‐dependent PL spectra from 80 to 380 K. e) FWHM and f) PL peak wavelength as a function of temperature derived from (d).

The as‐synthesized centimeter‐sized crystals are of sufficient quality and macroscopic dimensions to enable a detailed investigation of their optical properties, which were first investigated by UV‐vis absorption and PL spectra. **Figure** **2**a shows that the MA_2_CuCl_3_ single crystals lack a clear absorption edge in its UV‐vis spectrum, a strong sign that the crystals contain a certain number of sub‐band‐gap states which lie below its conduction band edge.^[^ ^31^ ^]^ The slope of this exponential part of the curve is the so‐called Urbach energy (*E*_U_).^[^ ^32^ ^]^ A large *E*_U_ reflects the semiconductors suffer from impurities, inherent structural disorders, and/or electron–phonon interaction in the absorption process.^[^ ^33^, ^34^, ^35^ ^]^ *E*_U_ value of the MA_2_CuCl_3_ single crystals was obtained by fitting their absorption coefficient *α*(*E*) versus photon energy *E* at a given temperature *T* (see inset of Figure 2a). According to Urbach rule, *α*(*E*) follows the relation^[^ ^36^ ^]^

(1) $$\alpha \left(E\right)=\alpha_{0}\exp\left[\sigma \left(T\right)\frac{E-E_{0}}{k_{B}T}\right]$$

where *α*_0_ and *E*_0_ are the characteristic parameters of the material, *σ*(*T*) is the steepness parameter and *K*_B_ is the Boltzmann constant. The quantity *K*_B_ *T*/*σ*(*T*), which is the width of the exponential tail, is *E*_U_. By fitting the band tail state, we obtained *E*_U_ value ≈84 meV, significantly larger than most of Pb‐based bulk perovskites (≈15 meV).^[^ ^37^, ^38^ ^]^ Such large *E*_U_ can be attributed to the effect of strong electron–phonon interaction in MA_2_CuCl_3_, which induces elastic structural distortion and creates transient electronic states upon photoexcitation. The strength of electron–phonon coupling in MA_2_CuCl_3_ will be studied in the following text using temperature‐dependent PL measurement. The PL excitation (PLE) spectrum shown in Figure S4 in the Supporting Information indicates that the MA_2_CuCl_3_ single crystals have an optimal excitation wavelength around 310 nm. Under 310 nm UV excitation, bulk crystals of MA_2_CuCl_3_ exhibited broadband warm white emission from 450 to 800 nm and had large Stokes shift of 200 nm, indicating negligible overlap between absorption and emission spectra (Figure 2b). This is of great importance for luminescent materials as one would expect minimum loss of light emission in thin films unlike excitonic or band edge emission.^[^ ^39^ ^]^ According to the Commission Internationale de l'Eclairage (CIE) 1931 standard color matching functions, the emitted light can be demonstrated by (*x*, *y*) chromaticity coordinates (0.44, 0.52) and a CCT of around 3607 K in the warm white region (Figure S5, Supporting Information). Remarkably, PLQY of these dopant‐free MA_2_CuCl_3_ single crystals reaches as high as 97%, making them one of the most efficient warm white‐light emitters reported to date. Table S7 in the Supporting Information summarizes and compares optical properties of some recently developed Cu^+^‐based hybrids, which visually showcases the uniqueness of our MA_2_CuCl_3_ as a warm white light‐emitting material. The high PLQY implies that the carrier recombination in such materials is dominated by radiative process and the defect‐related nonradiative recombination is negligible. This also supports our assumption that the large *E*_U_ of MA_2_CuCl_3_ originates from its strong electron–phonon interaction, rather than impurities or inherent structural disorders because the latter two scenarios would usually introduce nonradiative recombination centers that lower PLQY. To shed more light on how carriers in MA_2_CuCl_3_ crystals recombine through radiative process, time‐resolved PL (TRPL) measurements were conducted. Figure 2c shows the typical TRPL curve for MA_2_CuCl_3_ single crystals monitoring the emission peak at 567 nm. A good fit to the data was obtained by using a mono‐exponential decay function with lifetime constant of 50.45 µs. This result points to a single recombination channel in MA_2_CuCl_3_, which is in good line with their high PLQYs. It is noteworthy that PL lifetime of the MA_2_CuCl_3_ single crystals is much longer than most of the previously reported Pb‐free and Pb‐based perovskites whose PL lifetimes at room temperature range from tens of nanoseconds to several microseconds.^[^ ^6^, ^40^, ^41^, ^42^ ^]^ Such long radiative lifetime observed in MA_2_CuCl_3_ crystals, as we will discuss later, can be associated with their unique crystal structure, which spatially separates photo‐excited electrons and holes, giving rise to prolonged exciton lifetime.

To probe into the underlying mechanism of the extraordinary broadband emission of MA_2_CuCl_3_ single crystals, we then examined their emission wavelength‐dependent PLE spectra and excitation wavelength‐dependent PL spectra. It was found that both PLE and PL spectra exhibit identical shapes at various emission (from 500 to 660 nm) and excitation wavelengths (from 240 to 320 nm) (Figures S6 and S7, Supporting Information), indicating that the broadband emission originates from the relaxation of the same excited states and rules out the possibility of ion luminescence from Cu^+^.^[^ ^15^, ^43^, ^44^ ^]^ It is noteworthy that the presence of permanent defect states in semiconductors could also contribute to broadband emission. However, because permanent defect recombination lifetime and concentration are finite, their PL should be saturated at high excitation intensity.^[^ ^40^, ^45^ ^]^ To examine this, Figure S8 in the Supporting Information records the power‐dependent PL intensity of the MA_2_CuCl_3_ single crystals at room temperature. Linear relationship between the excited power and the intensity of PL peak was observed in the range of 2.2–10.5 mJ, thereby the possibility of permanent defects emission in MA_2_CuCl_3_ could be also excluded.

The distinct features, such as the broadband emission with high PLQY, large Stokes shift, and long excitonic lifetime, strongly suggest a self‐trapped exciton (STE) emission in MA_2_CuCl_3_. STEs are those photo‐excited electron–hole pairs whose coupling with lattice is strong enough to cause elastic distortion in the lattice surrounding them. As a result, transient, light‐induced trap states formed below the conduction band, affording a broad emission with a large Stokes shift.^[^ ^46^, ^47^, ^48^ ^]^ Recently, several other families of In^3+^‐, Cu^+^‐based halides and layered Sn, Pb halide perovskites were reported to exhibit the STE‐related emission.^[^ ^47^, ^49^, ^50^, ^51^, ^52^ ^]^ Interestingly, they all feature a strong electron–phonon coupling which results from their high structural distortion. To study the electron–phonon coupling in our MA_2_CuCl_3_ single crystals, Huang‐Rhys factor (*S*), which characterizes the strength of electron–phonon coupling, was extracted by using the following equation^[10]^

(2) $$\mathrm{FWHM}=2.36\sqrt{S}\hbar \omega_{\mathrm{photon}}\sqrt{\coth\frac{\hbar \omega_{\mathrm{photon}}}{2k_{B}T}}$$

where FWHM is the PL full‐width at half‐maximum and *ℏω*_phonon_ is the phonon frequency. Figure 2d shows the temperature‐dependent PL spectra of MA_2_CuCl_3_, where we see a noticeable increase in PL intensity from 80 to 340 K and then a rapid decrease above 360 K; at ≈380 K, MA_2_CuCl_3_ single crystals basically loss PL. It is noteworthy that unlike those all‐inorganic metal halide perovskites, such as CsCu_2_X_3_,^[^ ^24^ ^]^ Cs_3_Cu_2_X_5_,^[^ ^53^ ^]^ Rb_7_Sb_3_Cl_16_,^[^ ^54^ ^]^ Rb_2_CuBr_3_,^[^ ^55^ ^]^ and RbPbI_3_,^[^ ^56^ ^]^ whose PL emission consecutively decreases with increasing temperature, the positive correlation between PL emission and temperature observed in MA_2_CuCl_3_ in the range of 80 to 340 K is actually quite common in these organic–inorganic hybrid compounds, including Pb‐ and Sn‐based perovskites.^[^ ^51^, ^57^ ^]^ It is suggested that because the organic framework in these materials is rigid at low temperatures, it could be difficult for the crystals to induce a structural distortion and therefore difficult to create self‐trapped states. Besides, because of the low kinetic energy at low temperatures, it becomes difficult for the excitons to surmount potential barrier and to enter the self‐trapped states. Another possible mechanism for the enhanced STE emission with increasing temperature could be associated with the thermally activated delayed fluorescence, which involves inverse intersystem crossing from long‐lived triplet back to short‐lived singlet excited states.^[^ ^58^, ^59^, ^60^ ^]^ As for the rapid decrease of PL intensity from 340 to 380 K, it is probably due to the thermal quenching caused by the increased thermally populated vibrational states at high temperatures.^[^ ^61^ ^]^ Particularly, the dramatic PL quenching at 380 K is ascribed to the phase transition of MA_2_CuCl_3_ (from solid to liquid), as evidenced by DSC analysis. The FWHM data, derived from the temperature‐dependent PL spectra, were plotted in Figure 2e. Unlike the trend in PL intensity, FWHM changes monotonously with the increasing temperature. It broadens significantly from 80 to 360 K, indicative of an increased optical phonon scattering with temperature.^[^ ^62^, ^63^, ^64^ ^]^ By fitting FWHM curve using Equation (2), the *ℏω*_phonon_ is extracted as 20.5 ± 0.9 meV and the *S* factor is 41.9 ± 2.8. We note that the obtained *ℏω*_phonon_ value matches well with the other Cu^+^‐based organic–inorganic metal halides (20–40 meV), whereas *S* factor of MA_2_CuCl_3_ is obviously higher (vs 8.46 for (TBA)CuCl_2_, 10.7 for MA_2_CuBr_3_, 23.2 for [(C_3_H_7_)_4_N]_2_Cu_2_I_4_, and 11.95 for (C_16_H_36_N)CuI_2_).^[^ ^17^, ^18^, ^25^, ^61^ ^]^ This result indicates an exceptionally strong exciton–phonon coupling in MA_2_CuCl_3_, which can be also corroborated by their obvious blueshift of the PL band position with increasing temperature (Figure 2f) because at higher temperatures more optical phonons would participate in the exciton–phonon interaction through the short‐range deformation potential.^[^ ^65^, ^66^, ^67^ ^]^ Such strong exciton–phonon coupling paves the way for the formation of self‐trapped excited states and thus the ensuing broadband emission.

**Figure 3:**
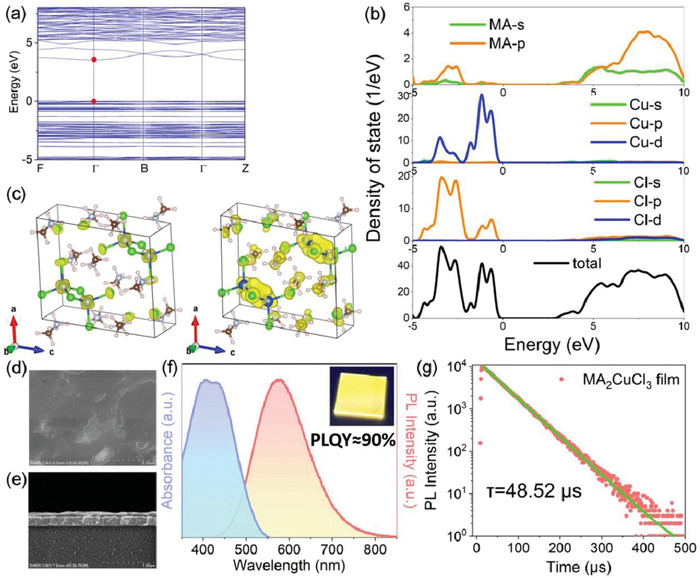
a) The calculated energy‐band structure and b) PDOS for MA_2_CuCl_3_. The zero of the energy was set at the top of the valence band. c) Diagram of the partial charge density for the VBM (left) and CBM (right) for the MA_2_CuCl_3_ crystal (yellow is electron cloud distribution, purple spheres are N, blue spheres are Cu, pink spheres are H, gray spheres are C, and green spheres are Cl atoms). d) Cross‐sectional and e) plane view SEM images, f) UV‐vis absorption, PL spectra, and g) time‐resolved PL decay curve of the as‐prepared MA_2_CuCl_3_ thin films on ITO glass. The inset of (f) shows pictures of a typical thin‐film sample taken under ambient light and 254 nm ultraviolet light, respectively.

To facilitate a better understanding on the electronic properties of MA_2_CuCl_3_ and to unveil the physical origins of their high emission efficacy, density functional theory (DFT) calculations were performed (computational details can be found in the Supporting Information). The calculated band structure of MA_2_CuCl_3_ shows a direct band gap of 3.47 eV at the Г point (**Figure** **3**a), which is in good agreement with the sharp absorption peak at 370 nm (≈3.35 eV, see Figure 2a). The projected density of states (PDOS) (Figure 3b) shows that the conduction band minimum (CBM) of MA_2_CuCl_3_ is composed mainly of MA‐s and MA‐p orbitals, whereas their valence band maximum (VBM) consists of Cu‐d, mixed with some Cl‐p orbitals, as indicated by the partial charge density (Figure 3c). The VBM of MA_2_CuCl_3_ features an almost flat and highly discrete band, which could be associated with the spatial isolation of [Cu_2_Cl_6_]^4–^ dimers by MA^+^ molecules, giving rise to a strong quantum confinement for the photo‐excited holes in MA_2_CuCl_3_. Because photo‐excited holes are highly localized in VB of the crystal, they will become less sensitive to defect states and hence contribute to enhanced radiative recombination and a single‐exponential PL decay.^[^ ^12^, ^13^, ^17^, ^55^ ^]^ Further, the analysis of the PDOS shows that the CBM of MA_2_CuCl_3_ mainly consists of MA^+^ orbitals and the VBM mainly comes from [Cu_2_Cl_6_]^4–^. Such spatial isolation of the two clusters also means that the photo‐excited holes and electrons are spatially separated, leading to a delayed charge recombination and thus a prolonged PL lifetime, in good line with the TRPL results which show exceptionally long‐lived excitons.

Stabilities of a luminescent material especially optical stabilities are of paramount importance for assessing their suitability for light‐emitting applications. Previous temperature‐dependent PL spectra of MA_2_CuCl_3_ single crystals reveal a significant increase in PL intensity when the samples were heated from 80 to 340 K and then a rapid decrease above 360 K; at ≈380 K, the material basically losses PL (Figure 2d). The rapid decrease in PL intensity above 360 K is because the single crystals undergo a melting process, which is, however, reversible. Figure S9 in the Supporting Information evidences the successful recovery of PL emission upon cooling from the liquid state, as well as the crystal structure. Storage stability of the MA_2_CuCl_3_ single crystals was next evaluated. Figure S10 in the Supporting Information shows that MA_2_CuCl_3_ single crystals retain 50% of the initial PL intensity after exposure for 4 days in ambient air (≈30% relative humidity, RH, 300 K), while negligible change in PL intensity was observed when they are stored in an inert atmosphere (100% N_2_) for over 6 months. To examine the impact of humidity on optical stability of the crystals, we have further put the sample in a high humidity atmosphere (98% RH, 300 K). PL spectra shown in Figure S11 in the Supporting Information indicate an accelerated decrease in emission intensity, which dropped below 50% of the initial value after 3 days of high humidity exposure. In addition to moisture attack, the gradual oxidation of Cu^+^ should be also held accountable for the decrease in PL intensity.^[^ ^68^, ^69^ ^]^ Overall, the above results suggest that for optimal utilization of MA_2_CuCl_3_, proper encapsulation will be required. Light stability of the MA_2_CuCl_3_ single crystals was also assessed as it is closely related to their down‐conversion phosphor applications. Figure S12 in the Supporting Information shows that under a continuous irradiation of 254 nm light, the crystal retains almost 70% of the initial intensity for over 24 h, which points to an excellent light stability.

To better utilize the intrinsically high PLQYs of MA_2_CuCl_3_ and to enhance their prospects and potential in LED applications, we demonstrated large‐scale and uniform thin films of MA_2_CuCl_3_ on indium‐doped tin oxide (ITO) glass. It should be noted that one of the most critical factors hindering the successful implementation of these Pb‐free halides in optoelectronics is the lack of a suitable method to process them into compact thin films. Previously, warm white‐emissive Cs_2_Ag*_x_* Na_1−_ *_x_* InCl_6_ thin films are thermally deposited by a vacuum‐based technique.^[^ ^6^ ^]^ Apart from the high cost and the general difficulty in precise stoichiometric control, the thermally deposited thin films are mainly consisted of those isolated nanograins with a broad size distribution. It is well known that an efficient LED device necessitates an efficient charge recombination in the emissive layer, too large a grain size or too thick an active layer will simply exacerbate problems of inefficient energy transfer, and thus deteriorate the device performance.^[^ ^70^, ^71^, ^72^ ^]^ Actually, for both Pb‐based metal halides and their derivatives, the solution‐based deposition technique has become the main method of choice for fabricating active layers with controllable thickness and morphology. However, solution processability of the Cs_2_Ag*_x_* Na_1−_ *_x_* InCl_6_ perovskite is largely limited by the poor solubility of AgCl in most organic solvents, including DMF and DMSO. By contrast, CuCl can be readily dissolved in these coordinating solvents. We therefore turn to seek solution‐based route to MA_2_CuCl_3_ thin films by dissolving MACl/CuCl powders or as‐prepared MA_2_CuCl_3_ crystals in DMF or DMSO following literature methods for Cs_3_Cu_2_I_5_/CsCu_2_I_3_.^[^ ^2^ ^]^ However, despite the success in growing isolated MA_2_CuCl_3_ single crystals as described before, solution‐phase deposition of high‐quality thin films from such a solvent was unsuccessful and led to discontinuous films with many small cracks (see Figure S13, Supporting Information). To address this issue, we have developed a novel synthetic route, where precursor solution was prepared by dissolving the as‐prepared MA_2_CuCl_3_ single crystals in methanol. Spin coating MA_2_CuCl_3_ methanol solution followed by drying at mild temperatures (<60 °C) allows the successful recovery of MA_2_CuCl_3_ solids on ITO substrate (more experimental details can be found in the Supporting Information). Cross‐sectional and plane view scanning electron microscopy (SEM) of a typical thin‐film sample revealed it to be ≈80 nm in thickness, which shows no major microscopic cracks or pinholes (Figure 3d,e). XRD pattern of the prepared thin films evidences the formation of high‐purity monoclinic‐phase MA_2_CuCl_3_ (Figure S14, Supporting Information), whose CIE color coordinates, CCT value, PL spectrum, and radiative lifetime are both like that of the single‐crystal structures (Figure 3f,g and Figure S15, Supporting Information). The inset of Figure 3f presents a typical photograph of the prepared thin film, which exhibits very stable warm white‐light emission under 254 nm UV light and high PLQYs approaching 90%.

**Figure 4:**
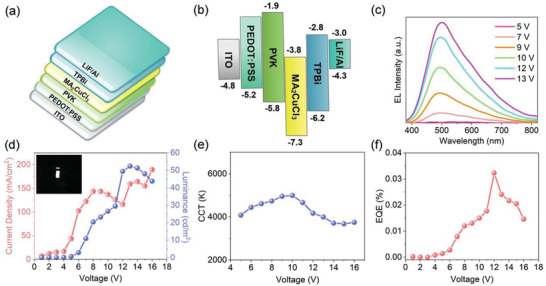
a) Device structure and b) energy band diagram (relative to the vacuum level) of MA_2_CuCl_3_ thin‐film‐based LEDs. c) EL spectra of the resulting LEDs at different applied voltages. d) *I*–*V*–*L* characteristics of MA_2_CuCl_3_ LED device. Inset: photograph of the LED driven by a 10 V bias. e) CCT values of the devices working under different voltages. f) EQE versus current density of these devices.

The successful deposition of such highly emissive and uniform thin films thus allows great flexibility in design and optimization of electronic and optoelectronic devices based on MA_2_CuCl_3_ material. To demonstrate this, we first prepared prototype electroluminescence (EL) devices using a traditional structure ITO/poly(3,4‐ethylenedioxythiophene):poly(styrene sulfonate) (PEDOT:PSS)/poly(9‐vinylcarbazole) (PVK)/MA_2_CuCl_3_/2,2′,2″(1,3,5‐benzenetriyl)*tris*‐(1‐phenyl‐1*H*‐benzimidazole) (TPBi)/LiF/Al on quartz, shown schematically in **Figure** **4**a, along with the band alignment in Figure 4b. Note that the valence band maximum of MA_2_CuCl_3_ was determined by ultraviolet photoelectron spectroscopy, shown in Figure S16 in the Supporting Information. However, despite intensive efforts including tuning MA_2_CuCl_3_ thin‐film thickness (50–100 nm), employing 1,3,5‐tri[(3‐pyridyl)‐phen‐3‐yl]benzene (TmPyPB) in place of TPBi, inserting a poly[*N*,*N*'‐bis(4‐butylphenyl)‐*N*,*N*'‐bis(phenyl)‐benzidine] (Poly‐TPD) or poly(9,9‐dioctylfluorene‐alt‐*N*‐(4‐butylphenyl)‐diphenylamine) (TFB) buffer layer before PVK, such device architecture cannot generate efficient EL, and due to the low luminous intensity, EL spectra of these devices cannot be displayed. The low luminous efficiency points to the presence of a significant injection barrier and/or charge injection imbalance inside the devices. We consider it possible given the extremely low valence band position of MA_2_CuCl_3_, which lies ≈1.5 eV below that of PVK and ≈2.1 eV with respect to PEDOT:PSS. Such large energy barrier between the hole injection layer (HIL) and emissive layer will bring difficulties in hole injection, and hence deteriorate the EL performance. To address this problem, we seek to lower valence band position of HIL by incorporating 20% 4,4'‐bis(carbazol‐9‐yl)biphenyl (CBP) into PVK. Excitingly, the CBP‐incorporated LEDs show noticeable white‐light emission at bias voltage from 7 to 13 V. Figure 4c presents EL spectra of the resulting LEDs at different applied voltages. Similar to MA_2_CuCl_3_ thin films, the EL showed very broad emission with FWHM ≈160 nm and the shape of the EL spectrum does not change at different applied voltages, signifying that the injected carriers are well confined in the emissive layers and the recombination zone does not change in response to different applied voltages. To our knowledge, this represents the first successful demonstration of LED application for this kind of Cu^+^‐based hybrid metal halide. It is noted that EL spectrum of the resulting LEDs underwent a blue shift (≈60 nm) with respect to the spectral line of MA_2_CuCl_3_ thin films, similar to observation of previously reported LEDs based on Cs_2_AgIn_0.9_Bi_0.1_Cl_6_ nanocrystals.^[^ ^73^ ^]^ However, unlike Cs_2_AgIn_0.9_Bi_0.1_Cl_6_ NC‐based LEDs, which exhibit an evident double‐color emission, there are no notable dual emission in MA_2_CuCl_3_ device, ruling out parasitic emission from the neighboring charge transport layers (e.g., PVK), as well as the undesired radiative recombination through band‐to‐band transitions. Figure 4d presents the current–voltage–luminance (*I*–*V*–*L*) characteristics of the MA_2_CuCl_3_ LEDs, with the upper inset shows digital photograph of the LED driven by a 10 V bias. The emitted light shows CIE color coordinates (0.35, 0.37) and CCT value of around 5000 K. Figure 4e records CCT values of the devices under different applied voltages. The turn‐on voltage (calculated at a luminance of 1 cd m^−2^) required for the LED device is ≈6 V. LEDs demonstrate a maximum brightness of 54 cd m^−2^ and a maximum EQE of 0.035% (Figure 4f). Such EL performance is slightly lower than that of the best‐performing white‐emissive Cs_2_AgIn_0.9_Bi_0.1_Cl_6_‐based LEDs (EQE 0.06–0.08%),^[^ ^71^ ^]^ suggesting there still exists significant injection barrier inside our present devices, partially due to the low carrier mobility of MA_2_CuCl_3_ film because of the 0D structure at the molecular level. Further increase in electrical parameters of the MA_2_CuCl_3_‐based LEDs can be expected through a more rational design of device architecture and optimization of charge‐transport layers. LED performance was maintained almost constant when the devices were stored in N_2_ atmosphere for 1 week, but dropped to ≈35% of the initial level after 12 h of storage in ambient air (RH 30–40%) (Figure S17, Supporting Information), which is inferior to that of the pure MA_2_CuCl_3_ single crystals (Figure S10, Supporting Information, *T*_50_ = 4 days). We consider it to be associated with their ultra‐thin thickness (≈80 nm), because of which the destructive effects of moisture attack would become more prominent. To further evaluate the operational stability of the ensuing LEDs, time‐dependent EL measurement was performed, without using epoxy or silica encapsulation for protection. It is seen that luminance of the resulting LEDs gradually decreased over time, which retained 50% of its initial intensity at *T* = 500 s (Figure S18, Supporting Information). Further study is required to clarify the possible degradation pathways and mechanisms under electric field strengths, with the aim of improving their operational stability.

**Figure 5:**
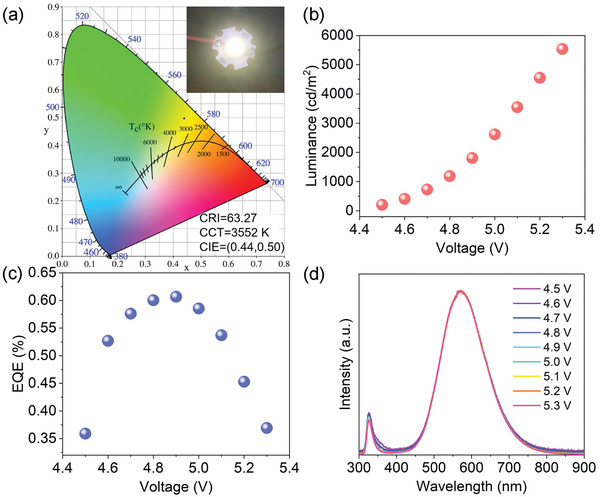
a) CIE coordinates and the main luminous parameters of the UV‐pumped MA_2_CuCl_3_ LEDs. Inset shows photograph of a typical device in operation. b) Luminous intensity, c) EQE, and d) normalized PL spectra of the fabricated warm‐WLEDs under different driving voltages.

We have also prepared UV‐pumped down‐conversion LEDs by directly pressing the MA_2_CuCl_3_ powders onto a commercial UV LED chip (310 nm), without using epoxy or silica encapsulation for protection. **Figure** **5**a shows the photograph of the as‐fabricated LEDs that emit bright and uniform warm white light, which features CIE color coordinates of (0.44, 0.50) and CCT of 3552 K in the warm white region. The device shows a brightness up to 5500 cd m^−2^ at a driving voltage of ≈5.3 V and highest EQE of 0.6% at ≈4.8 V (Figure 5b,c). Importantly, the emission color is high stability. Figure 5d shows the normalized PL spectra of the fabricated WLEDs, which remain almost constant when the voltage level is varied from 4.5 to 5.3 V. These results combined with the good CCT and color rendering index stability (Figure S19, Supporting Information) indicate that these Pb‐free organic–inorganic halides are also very promising for warm white‐light phosphor applications. To study the operational stability of the above UV‐pumped MA_2_CuCl_3_ LEDs, we further tracked the luminance of the unencapsulated devices at a driving voltage of ≈5 V. It is shown LEDs retain about 90% of the initial luminance after ≈45 min of operation in ambient air (RH ≈30%) but fall rapidly to 50% at *T* = 1 h. Such decline trend is found to have a direct correlation with the stability of the as‐bought 310 nm LED chips (Figure S20, Supporting Information). We foresee that a higher operational stability of the UV‐pumped MA_2_CuCl_3_ LEDs can be achieved by using a more stable UV chip.

## 3 Conclusions

This work provides a simple, low‐cost, and effective method for preparing centimeter‐level MA_2_CuCl_3_ single crystals and functional thin films, which represent a new kind of efficient warm white luminescent material based on organic–inorganic cuprous chloride. The observations of the broadband emission as well as the high PLQYs (≈97% without doping) in MA_2_CuCl_3_ single crystals were rationalized by means of temperature‐dependent PL measurements and DFT calculations, which unveil its exceptionally strong exciton–phonon coupling and unique spatial isolation of photo‐excited holes and electrons. Further, we developed a novel methanol‐based approach to deposit MA_2_CuCl_3_ thin films with uniform morphology and bright PL. Prototype EL devices and down‐conversion LEDs are fabricated with MA_2_CuCl_3_ thin films and single crystals, respectively, which show desirable warm white‐light emission yet still need significant improvement in efficiency to fulfill the requirements for indoor lighting. We foresee that our study will prompt future research on hybrid‐type Cu^+^‐based compounds and their use in single‐component warm‐WLEDs.

## Conflict of Interest

The authors declare no conflict of interest.

## Supporting information

Supporting Information

Supporting Information

## Data Availability

The data that support the findings of this study are available from the corresponding author upon reasonable request.
