# Supplementary material for: Organic–Inorganic Hybrid Cuprous‐Based Metal Halides for Warm White Light‐Emitting Diodes
Source: Adv Sci (Weinh). 2022 Sep 6;9(31):2203596. doi: 10.1002/advs.202203596 (PMC9631088; doi:10.1002/advs.202203596)
Supplement: Supplementary file 1 — Supporting Information [file ADVS-9-2203596-s001.pdf]

ADVANCED SCIENCE
Open Access

Supporting Information

for *Adv. Sci.*, DOI 10.1002/advs.202203596

Organic–Inorganic Hybrid Cuprous-Based Metal Halides for Warm White Light-Emitting Diodes

*Xuan Meng, Sujun Ji, Qiujie Wang, Xiaochen Wang, Tianxin Bai, Ruiling Zhang, Bin Yang, Yimeng Li, Zhipeng Shao, Junke Jiang, Ke-li Han and Feng Liu**

Supporting Information

Organic-Inorganic Hybrid Cuprous-Based Metal Halides for Warm White Light-Emitting Diodes

Xuan Meng,[1] Sujun Ji,[1] Qiujie Wang,[1] Xiaochen Wang,[1] Tianxin Bai,[1] Ruiling Zhang,[1] Bin Yang,[2] Yimeng Li,[3] Zhipeng Shao,[3] Junke Jiang,[4] Ke-li Han,[+,1,2] and Feng Liu*,[1]

[1]Institute of Molecular Sciences and Engineering, Institute of Frontier and Interdisciplinary Science, Shandong University, Qingdao 266237, P. R. China

[2]State Key Laboratory of Molecular Reaction Dynamics, Dalian Institute of Chemical Physics, Chinese Academy of Science, Dalian 116023, P. R. China

[3]Qingdao Institute of Bioenergy and Bioprocess Technology, Chinese Academy of Sciences, Qingdao, 266101 P. R. China

[4]Univ Rennes, ENSCR, CNRS, ISCR (Institut des Sciences Chimiques de Rennes)-UMR 6226, Rennes, France

[+]Deceased 17 March 2022

*E-mail: fenglau189@sdu.edu.cn

1. Experimental Details

1.1 Materials

Methylamine hydrochloride (MACl, 98.0%, Meryer Chemical), cuprous chloride (CuCl, ≥ 99.95%, Aladdin), concentrated hydrochloric acid (HCl, AR, Sinopharm Chemical), cuprous bromide (CuBr, 99.9%, Aladdin), hydrobromic acid (HBr, 48 wt.% in H_2O, Aladdin), hypophosphorus acid (H_3PO_2, 50 wt.% in H_2O, Aladdin), methylammonium bromide (MABr, 99.5%, Macklin), N,N-Dimethylformamide (DMF, 99.0%, Macklin), methanol (≥ 99.95%, Aladdin), poly(9-vinylcarbazole) (PVK, Mw = 10,000~100,000, Xi'an Polymer Light Technology Corp.), 4,4'-Bis(carbazol-9-yl)biphenyl (CBP, 99.9%, FEIYUBIO), 1,3,5-tri[(3-pyridyl)-phen-3-yl]benzene (TmPyPB, 98%, Xi'an Polymer Light Technology Corp.), 2,2′,2″(1,3,5-

benzenetriyl)tris-(1-phenyl-1H-benzimidazole) (TPBi, 99.5%, Aldrich). All chemicals were used as received without further purification.

1.2 Preparation of MA_2CuCl_3 single crystals

(a) Inverse temperature crystallization method: 12 mmol of MACl and 3 mmol of CuCl were dissolved in concentrated HCl (0.35 mL)/H_3PO_2 (0.18 mL). It was heated to 130 °C and then slowly cooled down to 10 °C in a thermostat at a cooling rate of 3 °C/min. After ~48 hours of growth, the crystals were taken out of the solution, washed by hexane and dried with a N_2 gun.

(b) Room-temperature solvent evaporation-induced crystallization: Synthesis was carried out according to the procedure of Zhang *et al.*[1] CuCl (1 mmol) and MACl (4 mmol) were dissolved in the mixture of DMF (1 mL) and H_3PO_2 (60 μL) by ultrasonication. Single crystals were obtained *via* slow evaporation at room temperature in a vacuum drying oven overnight. The obtained colorless crystals were washed by hexane and dried with N_2 gun.

1.3 Single-crystal XRD measurement

A suitable crystal was selected and placed on a Bruker APEX-II CCD diffractometer. The crystal was kept at 150 K during data collection. Using Olex2,[2] the structure was solved with the SHELXT[3] structure solution program using Intrinsic Phasing and refined with the SHELXL refinement package using Least Squares minimization.

1.4 Preparation of MA_2CuCl_3 thin films on ITO glass

In a glove box, ~0.05 g of as-prepared MA_2CuCl_3 single crystal was dissolved in 3 mL of methanol. After dissolution, it was spin-coated on a precleaned ITO glass at 3600 rpm for 30 s and then heated at 40 °C for 10 minutes.

1.5 Preparation of electrically driven MA_2CuCl_3 light-emitting diodes (LEDs)

The ITO-coated glass substrates were rinsed with deionized water followed by sonication in deionized water, acetone, and isopropanol, respectively, each for 15 min. The substrates were then dried at 125 ± 5 °C for 10 min and treated with UV ozone for 10 min before use. Thereafter, the aqueous solution of PEDOT:PSS (Clevios Al 4083) filtered through a 0.45 μm PTFE filter was spin-coated on the precleaned ITO glass at

a speed of 4500 rpm for 40 s and then annealed at 125 ± 5 °C for 30 min in the ambient conditions. 1 ml of chlorobenzene solution which contains 8 mg of PVK and 2 mg of CBP was spin-coated on PEDOT:PSS layer at 4000 rpm for 30 s, followed by annealing at 150 °C for 30 min in a glove box. Subsequently, thin films of MA_2CuCl_3 were deposited according to procedure described in section **1.4**. Then, a 40-nm-thick TPBi layer was deposited using a vacuum thermal evaporator. Finally, the cathode was fabricated by thermal evaporation of a LiF layer (1.0 nm), followed by an Al layer (100 nm). Each substrate is patterned to realize three devices, each with an active area of 0.08 cm^2. All the devices were stored and characterized under the ambient atmosphere without encapsulation.

1.6 Preparation of UV-pumped MA_2CuCl_3 LEDs

As-grown MA_2CuCl_3 single crystals were first grounded to powders and then pressed onto a 310 nm UV-LED chip to obtain UV-pumped LEDs.

1.7 Computational details

The first-principles calculations were carried out based on the density functional theory (DFT) as implemented in the Vienna *ab initio* simulation package (VASP).[4-5] The projector augmented wave (PAW) method[6] was used to represent the interactions of the core-valence electrons. Exchange and correlation potentials were treated in the generalized gradient approximation (GGA) of the Perdew Burke Ernzerhof (PBE).[7] A kinetic energy cutoff of 500 eV was used for the plane wave expansion. Monkhorst-Pack[8] k-point sampling method with densities of 0.2 $Å^{-1}$ was used for the Brillouin zone integration. The vdW correction was included through the DFT-D3 method[9] with Becke-Johnson damping. During structure relaxation, the convergence criteria for the total energy and force were 1.0 $\times 10^{-6}$ eV and 0.005 eV/Å. The GGA + U method was adopted to account for the strong correlation effects of the transition metal ions. The U value of 5.5 eV was added to the d orbital of Cu according to the literatures.[10]

1.8 Characterizations

Powder X-ray diffraction (XRD) measurements were performed on a Rigaku SmartLab 9 kW diffractometer equipped with Cu Kα radiation (Rigaku Corp., Japan). The

morphology and crystal structure of the prepared samples were characterized using a field-emission scanning electron microscopy (FESEM) (JEOL JSM-6340 F). UV-vis absorption spectra were acquired with a Shimadzu UV-2600i spectrophotometer. X-ray photoelectron spectroscopy (XPS) data were accumulated on a photoelectron spectrometer (Thermo Fisher ESCALAB Xi+). Photoluminescence (PL), photoluminescence quantum yield (PLQY), and time-resolved PL spectra were measured using a FLS1000 Edinburgh Instruments spectrofluorometer equipped with the integrating sphere. The inductively coupled plasma-mass spectrometry (ICP-MS) measurements were conducted using Perkin-Elmer ICP-MS NexION 300X. The valence band potential of the material was determined by ultraviolet photoelectron spectroscopy (UPS) (EscaLab 250Xi, Thermo, USA). LED performances were evaluated using a source meter (Keithley 2400, Keithley Instruments Inc.) and absolute EQE measurement system (C9920-12, Hamamatsu Photonics) with an optical fiber connected to a spectrometer (PMA-12, Hamamatsu Photonics).

Table S1. Crystal Data and Structure Refinement for MA_2CuCl_3 Single Crystal.

Empirical formula	$C_2H_{12}Cl_3CuN_2$
Formula weight	234.03
Temperature/K	150.0
Crystal system	monoclinic
Space group	P2/n
a/Å	9.6688(5)
b/Å	8.3137(4)
c/Å	11.1005(5)
α/°	90
β/°	100.256(3)
γ/°	90

Volume/Å^3	878.04(7)
Z	4
ρ_{calc}g/cm^3	1.770
μ/mm^{-1}	11.263
F(000)	472.0
Crystal size/mm^3	0.2 × 0.15 × 0.1
Radiation	Cu Kα (λ = 1.54178)
2Θ range for data collection/°	10.64 to 148.928
Index ranges	-12 ≤ h ≤ 11, -10 ≤ k ≤ 8, -13 ≤ l ≤ 13
Reflections collected	5286
Independent reflections	1765 [R_{int} = 0.0426, R_{sigma} = 0.0540]
Data/restraints/parameters	1765/0/78
Goodness-of-fit on F^2	1.148
Final R indexes [I>=2σ (I)]	R_1 = 0.0673, wR_2 = 0.1584
Final R indexes [all data]	R_1 = 0.0727, wR_2 = 0.1673
Largest diff. peak/hole / e Å^{-3}	0.98/-0.86

Table S2. Fractional Atomic Coordinates (×10^4) and Equivalent Isotropic Displacement Parameters (Å^2×10^3) for MA_2CuCl_3 Single Crystal. U_{eq} is Defined as 1/3 of the Trace of the Orthogonalized U_{IJ} Tensor.

**Atom**	***x***	***y***	***z***	**U(eq)**
Cu01	1831.1(5)	6927.4(7)	3667.0(5)	65.9(3)
Cl02	-561.0(7)	6806.5(8)	3638.1(6)	51.7(3)
Cl03	2500	8975.1(11)	2500	58.3(3)
Cl04	3211.9(8)	6727.1(9)	5602.4(6)	55.0(3)
Cl05	2500	4436.9(12)	2500	63.9(4)

Atom	*x*	*y*	*z*	U(eq)
N006	5867(3)	6988(3)	4255(3)	59.4(6)
N007	208(3)	7011(3)	6540(3)	53.8(6)
C008	6450(4)	8537(5)	4696(4)	65.2(8)
C009	352(4)	8769(4)	6619(3)	63.1(8)

Table S3. Anisotropic Displacement Parameters (Å^2×10^3) for MA_2CuCl_3 Single Crystal. The Anisotropic Displacement Factor Exponent Takes the Form: $-2\pi^2[h^2a^{*2}U_{11}+2hka^*b^*U_{12}+\ldots]$.

Atom	U_{11}	U_{22}	U_{33}	U_{23}	U_{13}	U_{12}
Cu01	53.2(4)	78.0(5)	66.0(4)	15.3(2)	9.1(3)	-3.55(19)
Cl02	46.9(5)	53.5(5)	55.4(5)	0.7(2)	10.5(3)	2.1(2)
Cl03	73.9(7)	45.7(5)	60.4(6)	0	25.7(5)	0
Cl04	50.2(5)	64.5(5)	50.5(5)	4.8(2)	9.3(3)	3.7(2)
Cl05	63.6(6)	48.0(6)	88.0(7)	0	35.3(5)	0
N006	63.5(16)	57.4(14)	60.7(14)	-2.6(10)	20.5(12)	-2.0(11)
N007	55.7(14)	49.8(12)	58.4(13)	1.1(9)	17.4(11)	-1.4(10)
C008	65.7(19)	56.3(16)	77(2)	-9.8(14)	22.9(16)	-1.2(14)
C009	70.9(19)	50.0(16)	68.0(18)	-4.4(13)	11.2(15)	4.0(14)

Table S4. Bond Lengths for MA_2CuCl_3 Single Crystal.

Atom	Atom	Length/Å		Atom	Atom	Length/Å
Cu01	Cl02	2.3096(9)		Cu01	Cl05	2.5849(10)
Cu01	Cl03	2.3006(8)		N006	C008	1.455(4)

Atom	Atom	Length/Å		Atom	Atom	Length/Å
Cu01	Cl04	2.3248(9)		N007	C009	1.469(4)

Table S5. Bond Angles for MA_2CuCl_3 Single Crystal.

Atom	Atom	Atom	Angle/°		Atom	Atom	Atom	Angle/°
Cl02	Cu01	Cl04	114.75(3)		Cl03	Cu01	Cl05	100.95(3)
Cl02	Cu01	Cl05	107.31(3)		Cl04	Cu01	Cl05	104.59(3)
Cl03	Cu01	Cl02	113.93(3)		Cu01[1]	Cl03	Cu01	84.54(4)
Cl03	Cu01	Cl04	113.61(3)		Cu01[1]	Cl05	Cu01	73.55(3)

Symmetry transformations used to generate equivalent atoms:

[1]1/2-X,+Y,1/2-Z

Table S6. Hydrogen Atom Coordinates (Å×10^4) and Isotropic Displacement Parameters (Å^2×10^3) for MA_2CuCl_3 Single Crystal.

Atom	*x*	*y*	*z*	U(eq)
H00A	5723.09	6973.77	3422.58	71
H00B	5035.67	6829.05	4513.14	71
H00C	6479.21	6191.69	4554.24	71
H00G	-103.88	6720.56	5747.42	65
H00H	-417.68	6677.97	7009.77	65
H00I	1058.32	6545.2	6812.31	65
H00D	7325.68	8727.98	4389.85	98
H00E	6642.88	8535.57	5593.61	98

**Atom**	***x***	***y***	***z***	**U(eq)**
H00F	5774.21	9389.82	4403.07	98
H00J	963.67	9136.18	6059.31	95
H00K	763.44	9074.53	7458.94	95
H00L	-576.44	9267.39	6391.67	95

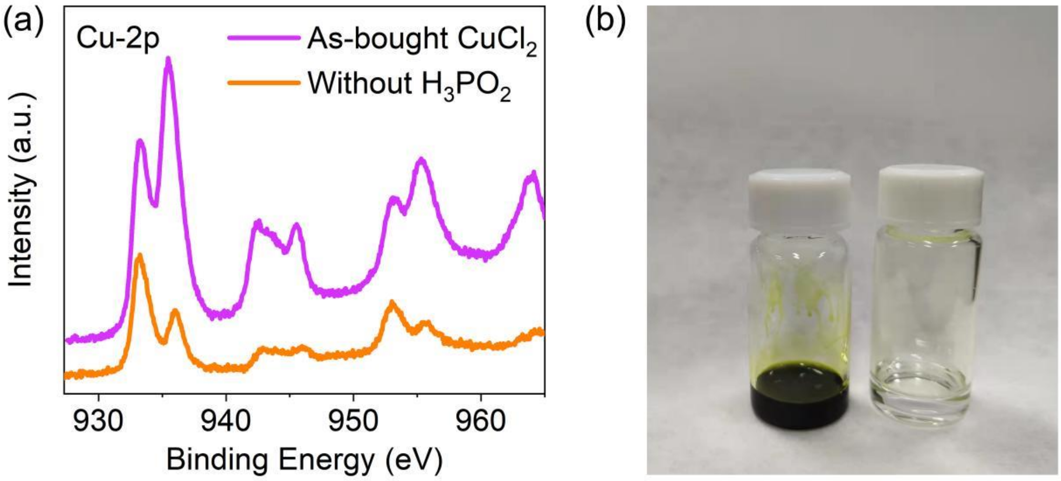

Figure S1. (a) Cu 2p XPS spectra taken from the as-bought $CuCl_2$ powder and solid product which was crystallized from MACl/CuCl solution (in HCl), but without the use of H_3PO_2. The binding energy was calibrated with the C 1s peak of free carbon (284.5 eV). (b) Photographs of two bottles of HCl solution which contains MACl and CuCl in the absence of H_3PO_2 (left) and with the addition of H_3PO_2 (right).

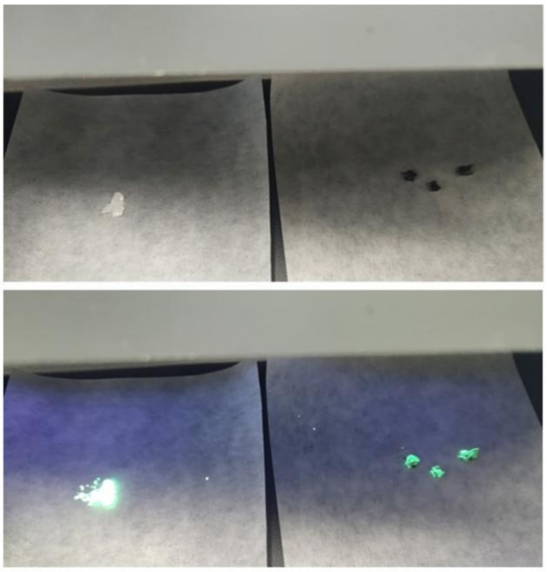

Figure S2. Photographs of solid products which were crystallized from MABr (12 mmol)/CuBr (3 mmol) solution (in concentrated HBr, 0.35 mL) (a) with and (b) without the use of H_3PO_2 (0.18 mL), respectively. It is seen that dark green solid products can be produced if we do not put H_3PO_2 in growth solution. These dark green solid products exhibit faint green PL, which is in stark contrast to the products obtained with the use of H_3PO_2.

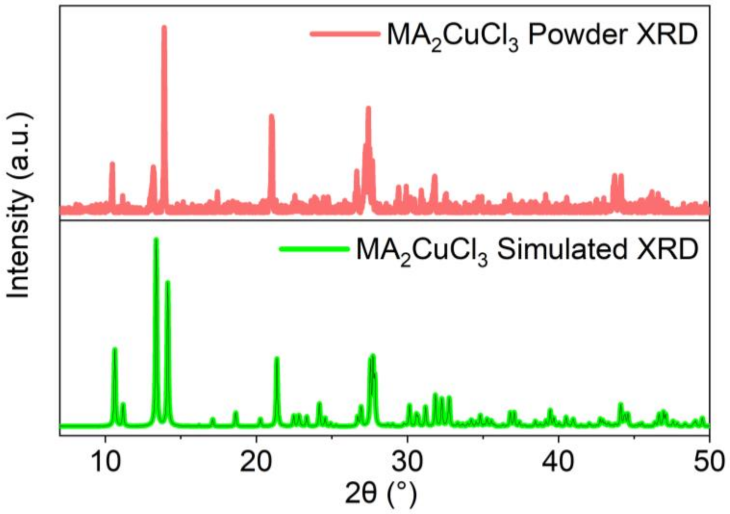

Figure S3. XRD pattern (red line) of the hand-ground powders which were obtained by room-temperature solvent evaporation-induced crystallization strategy. XRD pattern (yellow line) of the standard monoclinic-phase MA_2CuCl_3 was provided for comparison.

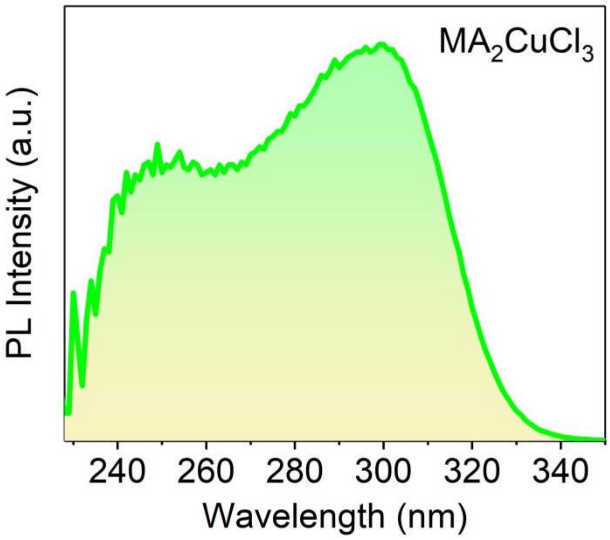

Figure S4. PL excitation (PLE) spectrum of the MA_2CuCl_3 single crystals.

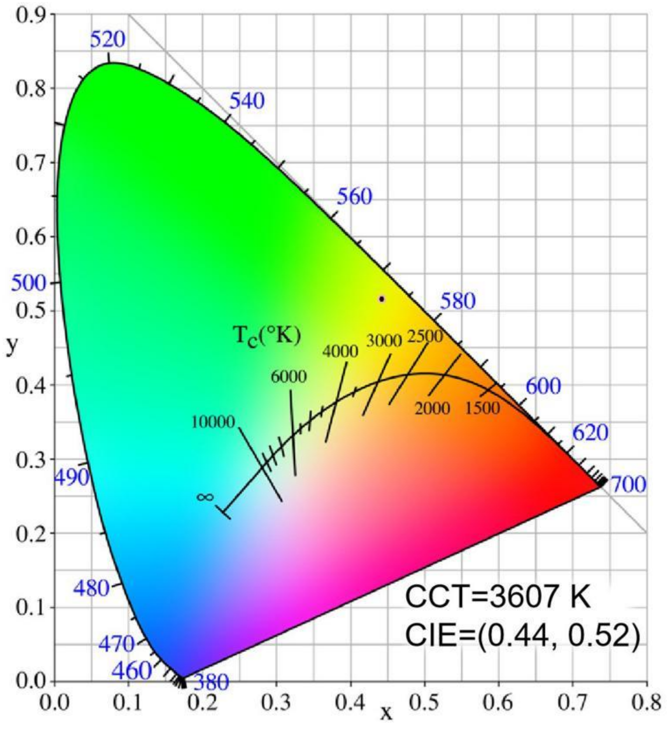

Figure S5. CIE coordinates and CCT value of the MA_2CuCl_3 single crystals.

Table S7. Optical properties of some recently developed Cu^+-based hybrids.

Cu^+-based hybrids	Emission peak (nm)	PL spectrum (nm)	PLQY (%)	color	CCT (K)	Ref.
$(MA)_4Cu_2Br_6$	524	400-700	93	green	6700	11
$TBACuCl_2$ (TBA = tetrabutylammonium)	510	395-630	92.8	green	9800	1, 12
$TBACuBr_2$	498	385-630	80.5	sky-blue	12000	1, 13
$TBA_2Cu_2I_4$	473、699	400-800	72.5	cool-white	8700	14
$TEA_2Cu_2Br_4$ (TEA = tetraethylammonium)	463	350-550	97.08	blue	\	15
$(DTA)_2Cu_2I_4$ (DTA = dodecyl trimethyl)	540	400-700	60	green-yellow	~5000	16

$(Bmpip)_2Cu_2Br_4$ (Bmpip = 1-butyl-1-methylpiperidinium)	620	450-800	48.2	orange	\	17
PPh_4CuBr_2 (PPh_4 = tetraphenylphosphonium)	538	450-750	1.3	yellow	\	17
$(Gua)_3Cu_2I_5$ (cool) (Gua = guanidine)	481	375-700	96	sky-blue	12500	18
$(Gua)_3Cu_2I_5$ (heated) (Gua = guanidine)	570	450-700	\	warm-white	~3800	18
$[(C_3H_7)_4N]_2Cu_2I_4$	483、637	400-800	91.9	cool-white	5684	19
$(C_{16}H_{36}N)CuI_2$	476、675	400-850	54.3	cool-white	~8000	20
$(TPA)CuCl_2$ (TPA = tetrapropylammonium)	505	420-600	91.8	green	\	21
MA_2CuCl_3	567	450-800	97	warm-white	3607	this work

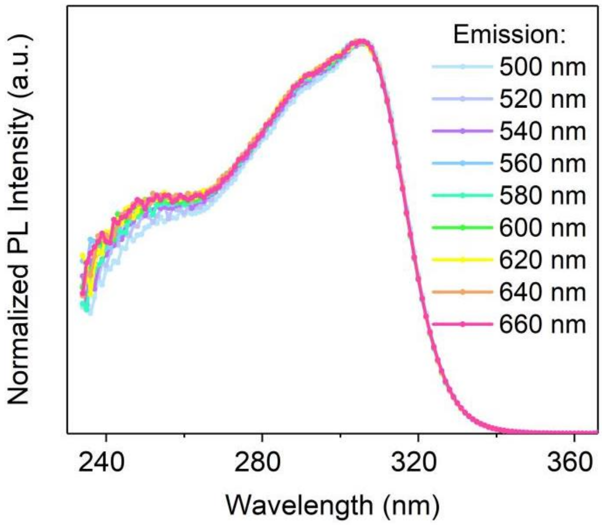

Figure S6. Emission wavelength-dependent PLE spectra of the MA_2CuCl_3 single crystals.

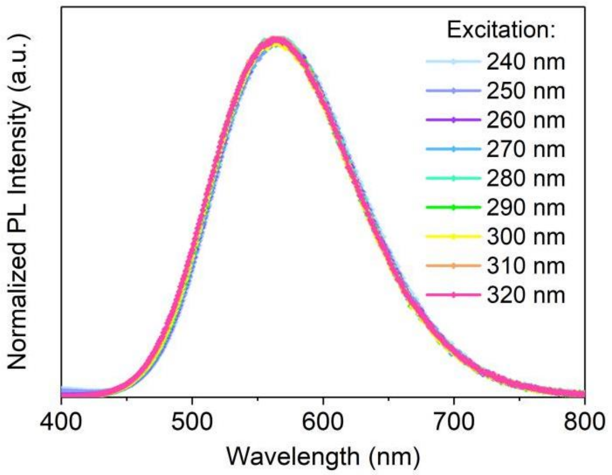

Figure S7. Excitation wavelength-dependent PL spectra of the MA_2CuCl_3 single crystals.

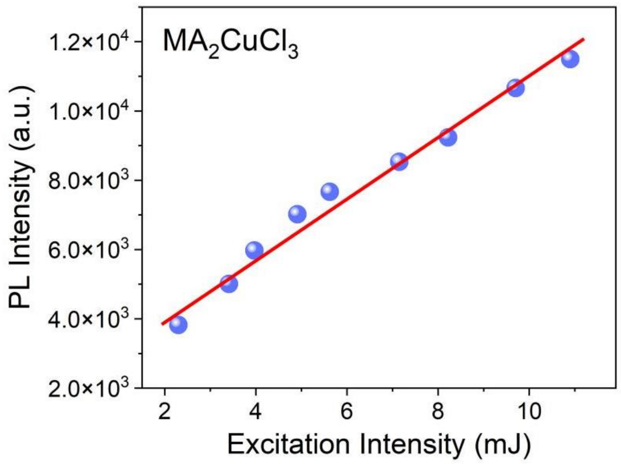

Figure S8. Power dependence of the intensity of the PL spectrum of the MA_2CuCl_3 single crystals at room temperature.

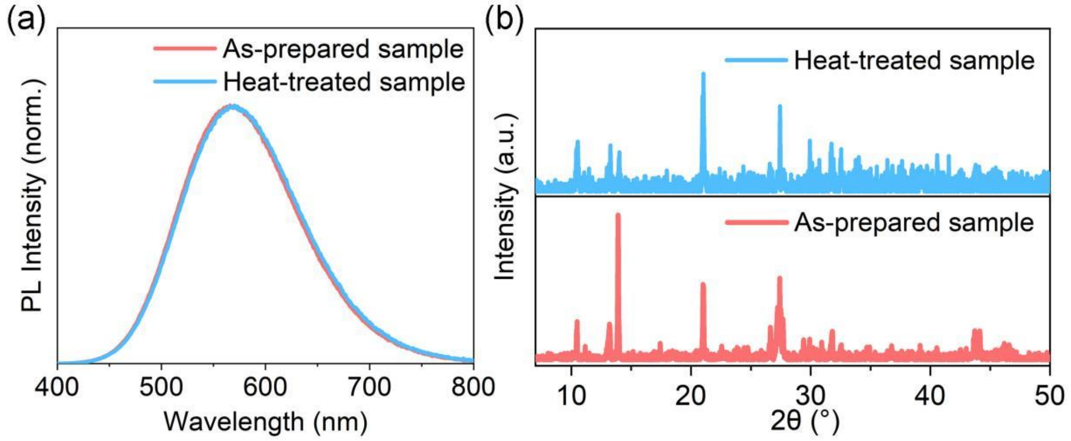

Figure S9. (a) PL spectra and (b) XRD patterns of the as-prepared MA_2CuCl_3 single crystals and those recovered from a melting process at 120 °C.

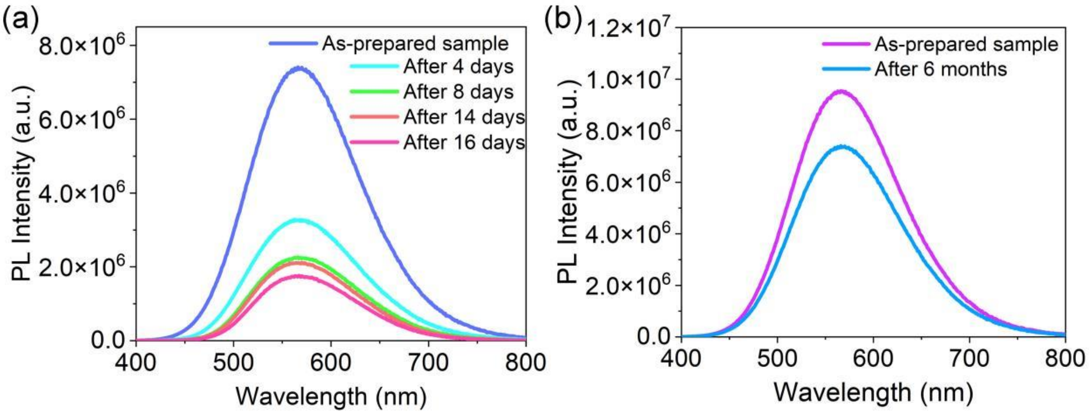

Figure S10. PL spectra of the MA_2CuCl_3 single crystals (a) after exposure in ambient air and (b) in an inert atmosphere for a period of time.

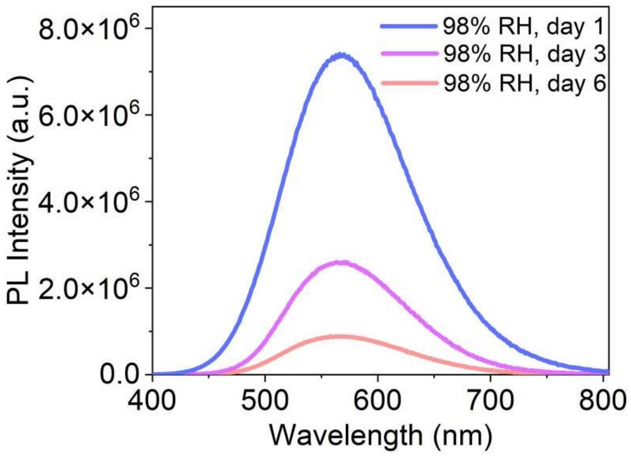

Figure S11. PL spectra of the MA_2CuCl_3 single crystals recorded at different time scales. The samples were exposed to high humidity (98% RH, 300 K).

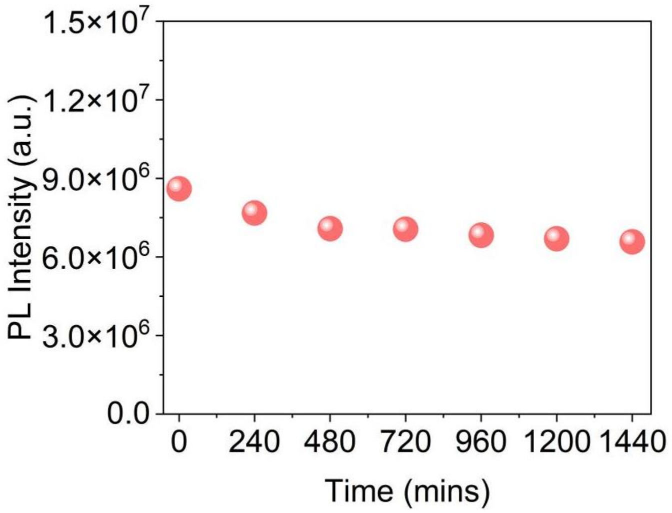

Figure S12. Time-resolved PL intensity (monitored at 567 nm) of the MA_2CuCl_3 single crystals with continuous irradiation of 254-nm light for 1 day. Note that samples were stored in a glove box (N_2 atmosphere) for irradiation.

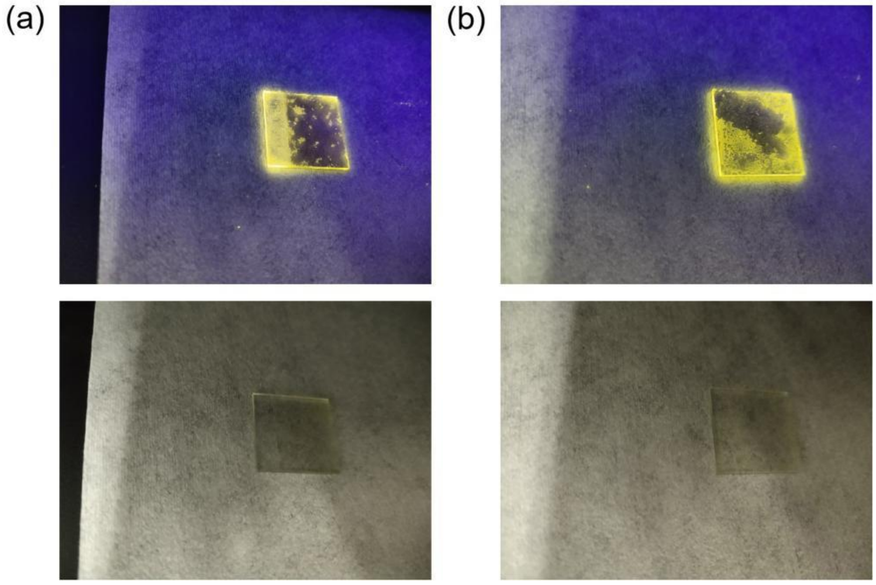

Figure S13. Photographs of thin film samples taken under ambient light and 254-nm UV light. Samples were prepared by spin-coating (a) DMF and (b) DMSO solutions of MACl/CuCl (molar ratio 4:1, with the addition of H_3PO_2) on ITO glass, followed by drying at 50 °C under N_2 atmosphere.

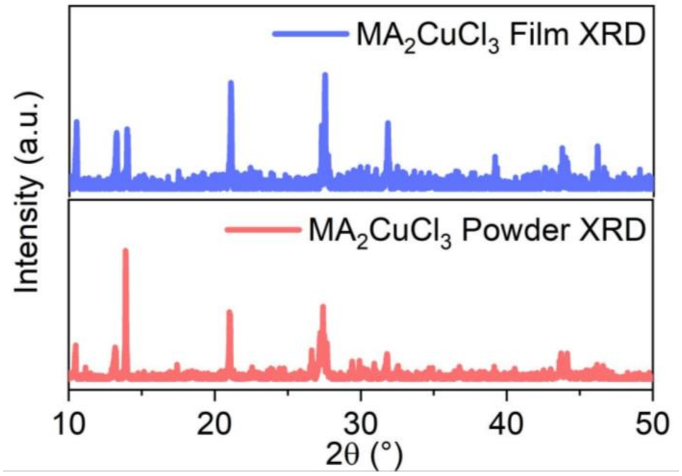

Figure S14. XRD pattern (blue line) of the prepared thin films by methanol method. XRD pattern (red line) of the prepared MA_2CuCl_3 single crystal powder was provided for comparison.

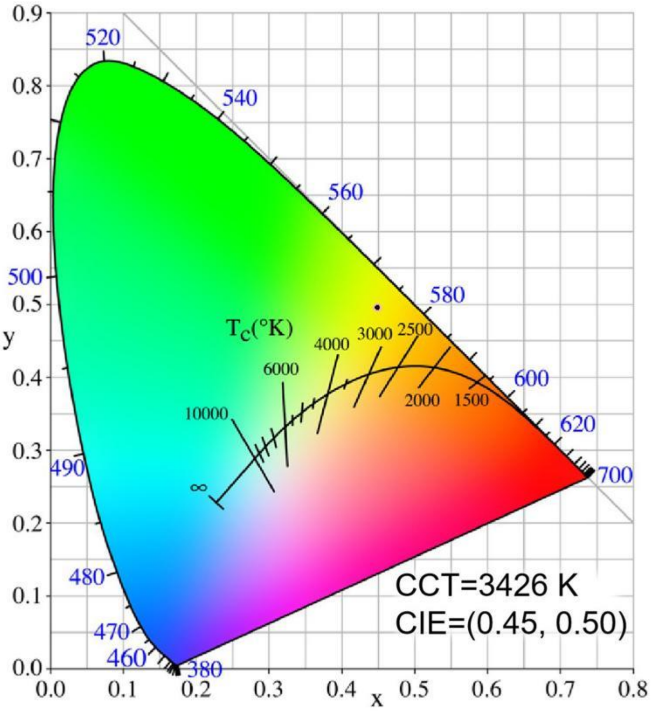

Figure S15. CIE color coordinates and CCT value of the as-prepared MA_2CuCl_3 thin films.

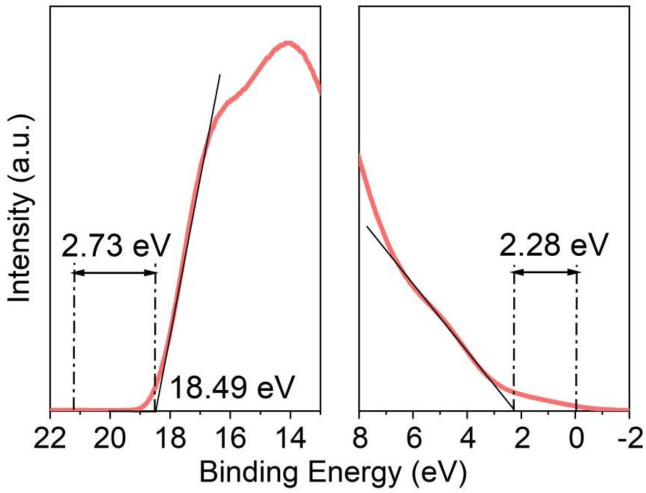

Figure S16. UPS spectrum of the MA_2CuCl_3 thin films. UPS spectrum was recorded using unfiltered He I excitation (21.22 eV) as the light source with the samples biased at -3.0 eV. The extrapolation of the linear portion in the spectrum represents the positions of secondary electron cut-offs and the valence edges, indicated as upward and downward wedge marks, respectively. Spectrum shown was displayed using the binding energy for the abscissas, scaled from the Fermi level, which was determined from the spectrum of a thick Au film. From the secondary electron cut-off region, the work-function of MA_2CuCl_3 is estimated to be 21.22 - (18.49 - 2.28) = 5.01 eV. From the valence band edge region, the energy separated between the valence band level and the Fermi level is determined to be 2.28 eV and thus the valence band maximum (VBM) is about -5.01 - 2.28 = -7.29 eV. The band gap of MA_2CuCl_3 is 3.47 eV which was determined from the absorption spectrum as shown in Figure 2a. Therefore, the conduction band minimum (CBM) is estimated to be -7.29 + 3.47 = -3.82 eV.

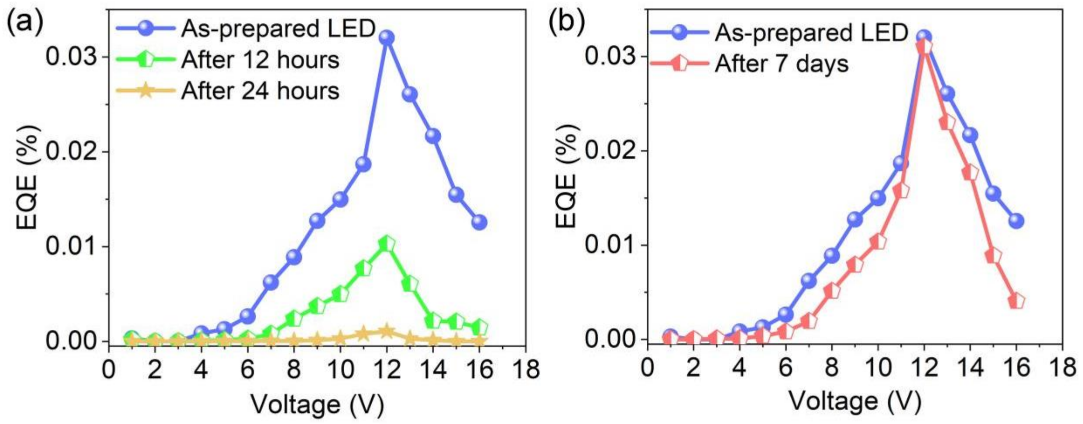

Figure S17. EQE of the MA_2CuCl_3-based LEDs after storage in (a) ambient air for 1 day and (b) in N_2 atmosphere for 7 days.

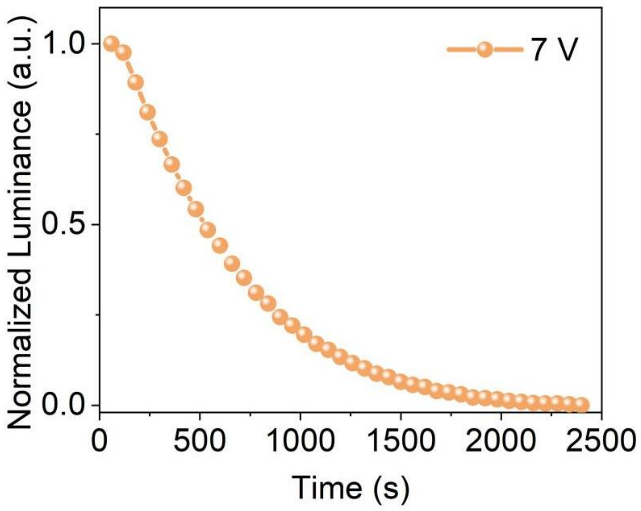

Figure S18. Luminance evolution of the fabricated LED devices working under constant bias voltage of 7 V (RH 30~40%, 30 °C).

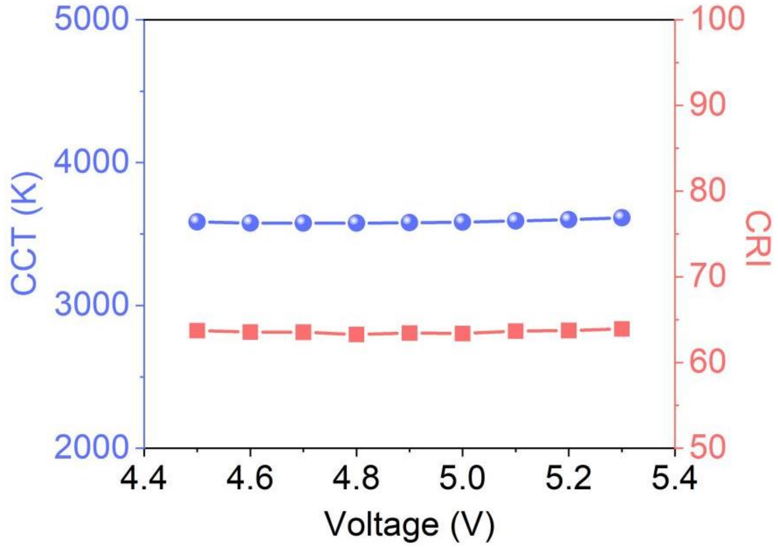

Figure S19. CCT and CRI of UV-pumped MA_2CuCl_3 LEDs under different driving voltages.

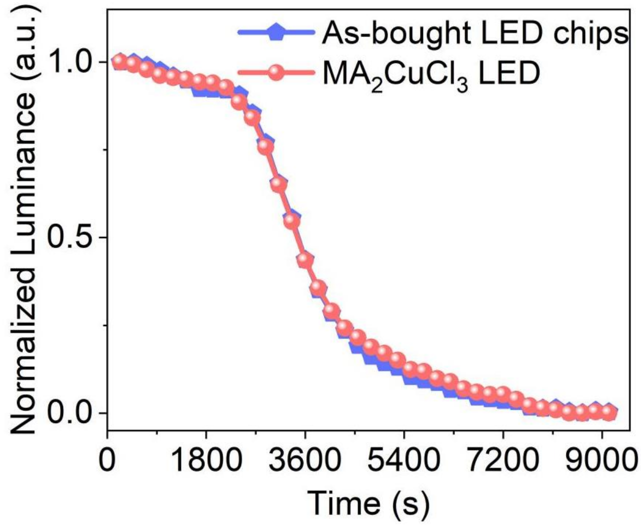

Figure S20. Time-dependent luminance of the unencapsulated UV-pumped MA_2CuCl_3 LEDs and the as-bought 310-nm LED chips (RH 30~40%, 30 °C).

References

[1] Lian, L.; Wang, X.; Zhang, P.; Zhu, J.; Zhang, X.; Gao, J.; Wang, S.; Liang, G.; Zhang, D.; Gao, L.; Song, H.; Chen, R.; Lan, X.; Liang, W.; Niu, G.; Tang, J.; Zhang, J., Highly Luminescent Zero-Dimensional Organic Copper Halides for X-ray Scintillation. *J. Phys. Chem. Lett.* **2021,** *12*, 6919-6926.

[2] Dolomanov, O. V.; Bourhis, L. J.; Gildea, R. J.; Howard, J. A.; Puschmann, H., OLEX2: A Complete Structure Solution, Refinement and Analysis Program. *J. Appl. Crystallogr.* **2009,** *42*, 339-341.

[3] Sheldrick, G. M., Crystal Structure Refinement with SHELXL. *Acta Crystallogr. C.* **2015,** *71*, 3-8.

[4] Kresse, G.; Furthmüller, J., Efficiency of ab-Initio Total Energy Calculations for Metals and Semiconductors using a Plane-Wave Basis Set. *Comp. Mater. Sci.* **1996,** *6*, 15-50.

[5] Kresse, G.; Joubert, D., From Ultrasoft Pseudopotentials to the Projector Augmented-Wave Method. *Phys. Rev. B* **1999,** *59*, 1758.

[6] Blöchl, P. E., Projector Augmented-Wave Method. *Phys. Rev. B* **1994,** *50*, 17953.

[7] Perdew, J. P.; Burke, K.; Ernzerhof, M., Generalized Gradient Approximation Made Simple. *Phys. Rev. Lett.* **1996,** *77*, 3865.

[8] Pack, J. D.; Monkhorst, H. J., " Special Points for Brillouin-Zone Integrations"—a Reply. *Phys. Rev. B* **1977,** *16*, 1748.

[9] Grimme, S., Semiempirical GGA-Type Density Functional Constructed with a Long-Range Dispersion Correction. *J. Comput. Chem.* **2006,** *27*, 1787-1799.

[10] Živković, A.; de Leeuw, N. H., Exploring the Formation of Intrinsic p-Type and n-Type Defects in CuO. *Phys. Rev. Mater.* **2020,** *4*, 074606.

[11] Peng, H.; Yao, S.; Guo, Y.; Zhi, R.; Wang, X.; Ge, F.; Tian, Y.; Wang, J.; Zou, B., Highly Efficient Self-Trapped Exciton Emission of a (MA)4Cu2Br6 Single Crystal. *J. Phys. Chem. Lett.* **2020,** *11*, 4703-4710.

[12] Peng, H.; Wang, X.; Tian, Y.; Dong, T.; Xiao, Y.; Huang, T.; Guo, Y.; Wang, J.; Zou, B., Water-Stable Zero-Dimensional (C4H9)4NCuCl2 Single Crystal with Highly Efficient Broadband Green Emission. *J. Phys. Chem. Lett.* **2021,** *12*, 6639-6647.

[13] Peng, H.; Tian, Y.; Zhang, Z.; Wang, X.; Huang, T.; Dong, T.; Xiao, Y.; Wang, J.; Zou, B., Bulk Assembly of Zero-Dimensional Organic Copper Bromide Hybrid with Bright Self-Trapped Exciton Emission and High Antiwater Stability. *J. Phys. Chem. C* **2021,** *125*, 20014-20021.

[14] Peng, H.; Xiao, Y.; Tian, Y.; Wang, X.; Huang, T.; Dong, T.; Zhao, Y.; Wang, J.; Zou, B., Dual Self-Trapped Exciton Emission of (TBA)2Cu2I4: Optical Properties and High Anti-Water Stability. *J. Mater. Chem. C* **2021,** *9*, 16014-16021.
[15] Liu, X.; Yuan, F.; Zhu, C.; Li, J.; Lv, X.; Xing, G.; Wei, Q.; Wang, G.; Dai, J.; Dong, H.; Xu, J.; Jiao, B.; Wu, Z., Near-Unity Blue Luminance from Lead-Free Copper Halides for Light-Emitting Diodes. *Nano Energy* **2022,** *91*, 106664.
[16] Liu, F.; Mondal, D.; Zhang, K.; Zhang, Y.; Huang, K.; Wang, D.; Yang, W.; Mahadevan, P.; Xie, R., Zero-Dimensional Plate-Shaped Copper Halide Crystals with Green-Yellow Emissions. *Mater. Adv.* **2021,** *2*, 3744-3751.
[17] Xu, T.; Li, Y.; Nikl, M.; Kucerkova, R.; Zhou, Z.; Chen, J.; Sun, Y.-Y.; Niu, G.; Tang, J.; Wang, Q.; Ren, G.; Wu, Y., Lead-Free Zero-Dimensional Organic-Copper(I) Halides as Stable and Sensitive X-ray Scintillators. *ACS Appl. Mater. Inter.* **2022,** *14*, 14157-14164.
[18] Peng, H.; Wang, X.; Tian, Y.; Zou, B.; Yang, F.; Huang, T.; Peng, C.; Yao, S.; Yu, Z.; Yao, Q.; Rao, G.; Wang, J., Highly Efficient Cool-White Photoluminescence of (Gua)3Cu2I5 Single Crystals: Formation and Optical Properties. *ACS Appl. Mater. Inter.* **2021,** *13*, 13443-13451.
[19] Peng, H.; Tian, Y.; Wang, X.; Huang, T.; Yu, Z.; Zhao, Y.; Dong, T.; Wang, J.; Zou, B., Pure White Emission with 91.9% Photoluminescence Quantum Yield of [(C3H7)4N]2Cu2I4 out of Polaronic States and Ultra-High Color Rendering Index. *ACS Appl. Mater. Inter.* **2022,** *14*, 12395-12403.
[20] Lian, L.; Zhang, P.; Liang, G.; Wang, S.; Wang, X.; Wang, Y.; Zhang, X.; Gao, J.; Zhang, D.; Gao, L.; Song, H.; Chen, R.; Lan, X.; Liang, W.; Niu, G.; Tang, J.; Zhang, J., Efficient Dual-Band White-Light Emission with High Color Rendering from Zero-Dimensional Organic Copper Iodide. *ACS Appl. Mater. Inter.* **2021,** *13*, 22749-22756.
[21] Peng, H.; Tian, Y.; Wang, X.; Dong, T.; Yu, Z.; Xiao, Y.; Zhang, Z.; Wang, J.; Zou, B., Highly Efficient Broadband Green Emission of (TPA)CuCl2 Single Crystals: Understanding the Formation of Self-Trapped States. *J. Phys. Chem. C* **2022,** *126*, 8545-8552.
